# Dietary Sources of Fiber Intake and Its Association with Socio-Economic Factors among Flemish Preschool Children

**DOI:** 10.3390/ijms12031836

**Published:** 2011-03-10

**Authors:** Yi Lin, Selin Bolca, Stefanie Vandevijvere, Willem De Keyzer, Herman Van Oyen, John Van Camp, Guy De Backer, Stefaan De Henauw, Inge Huybrechts

**Affiliations:** 1 Unit Nutrition and Food Safety, Department of Public Health, Faculty of Medicine and Health Sciences, Ghent University, De Pintelaan 185, B-9000 Ghent, Belgium; E-Mails: yi.lin@ugent.be (Y.L.); guy.debacker@ugent.be (G.D.B.); stefaan.dehenauw@ugent.be (S.D.H.); 2 Laboratory for Bioinformatics and Computational Genomics (BIOBIX), Faculty of Bioscience Engineering, Ghent University, Coupure Links 653, B-9000 Ghent, Belgium; E-Mail: selin.bolca@ugent.be; 3 Unit of Epidemiology, Scientific Institute of Public Health, J. Wytsmanstraat 14, B-1050 Brussels, Belgium; E-Mails: stefanie.vandervijvere@wiv-isp.be (S.V.); herman.vanoyen@wiv-isp.be (H.V.O.); 4 Department of Nutrition and Dietetics, Faculty of Health Care Vesalius, University College Ghent, Keramiekstraat 80, B-9000 Ghent, Belgium; E-Mail: willem.dekeyzer@hogent.be (W.D.K.); 5 Department of Food Safety and Food Quality, Ghent University, Coupure Links 653, B-9000 Ghent, Belgium; E-Mail: john.vancamp@ugent.be

**Keywords:** dietary fiber intake, preschool children, socio-economic status, Belgium

## Abstract

The objectives were to assess total dietary fiber intake, identify the major sources of dietary fiber, and examine its association with socio-economic factors among Flemish preschoolers. Three-day estimated dietary records were collected from a representative sample of preschoolers 2.5–6.5 years old (*n* = 661; 338 boys, 323 girls). The mean dietary fiber intake (13.4 g/d) was lower than the intake level recommended by the Belgian Superior Health Council (70% boys and 81% girls below the guidelines). The most important contributor was the group of bread and cereals (29.5%), followed by fruits (17.8%), potatoes and grains (16.0%), energy-dense, low-nutritious foods (12.4%), and vegetables (11.8%). Multiple linear regression analyses showed that total fiber intake was associated with maternal education and parents’ employment. Overall, fiber intakes from high-nutritious foods (vegetables and fruits) were higher in preschoolers of higher educated mothers and those with one or both parents being employed. In conclusion, the majority of the preschoolers had dietary fiber intakes below the recommended level. Hence, dietary fiber should be promoted among parents of preschoolers and low socio-economic status families should be addressed in particular.

## 1. Introduction

A significantly decreased dietary fiber (DF) intake and concomitant increased intake of total fat, saturated fatty acids and cholesterol in industrialized countries was found to be associated with a higher prevalence of chronic diseases [1,2]. The World Health Organization (WHO) identified a low DF intake as an important determinant for chronic diseases, including obesity, cardiovascular diseases, and diabetes [3]. DF is one of the nutritional compounds of vegetables, fruits, legumes, nuts, and whole-grain foods, known as carbohydrate polymers with ten or more monomeric units, that are not hydrolyzed by endogenous enzymes in the small intestine [4].

Evidence shows that a higher intake of DF is significantly associated with lower BMI, systolic and diastolic blood pressure, serum LDL-cholesterol and triglycerides [5–9]. Hence, a sufficient intake of DF is strongly recommended by the Belgian Superior Health Council (BSHC) [10], World Health Organization (WHO) [11], US Department of Agriculture (USDA) [12], and British Nutrition Foundation [13]. Many chronic diseases and some cancers in adults have been related to dietary factors during early childhood [14,15]. Williams and Bollella (1995) reported that a higher DF intake may have a positive effect on serum vitamin and mineral concentrations in healthy children consuming a balanced diet containing adequate levels of nutrients [16].

In addition, as dietary habits are established in early life, young children need to be encouraged to consume nutritious, fiber-rich foods daily to achieve an optimum health status [16]. DF intake of European preschoolers is poorly documented. Reported DF intakes in children and adolescents range from 0.9 to 3.5 g/MJ, although, different analytical methods or definitions were used [17]. A recent small-scale Flemish study (115 children, 2–3 y) [18] reported that the DF intake of children did not reach the recommendations of the BSHC [10]. As far as we are aware, no previous study has undertaken a comprehensive analysis of the food sources of DF among Belgian preschoolers. Furthermore, children and adolescents from low socio-economic status (SES) families were previously found to consume less DF, but higher energy-dense foods with higher risk of overweight and obesity [19–23]. Due to lack of knowledge on DF consumption in Belgian preschoolers, the present study aimed to assess the DF intake, and to indentify the major food sources of DF among Flemish preschoolers. Furthermore, the association between total and food group-specific fiber intakes and SES was examined.

## 2. Methods

### 2.1. Survey Population

This study used data from the Flanders preschool dietary survey (data collected from October 2002 until February 2003), in which the usual dietary intake of Flemish preschoolers (2.5–6.5 y) was estimated from 3-day estimated dietary records (EDR), completed by the parents. The distribution of 3d EDR covered a whole week in autumn and winter. The sampling design and methods have been described in detail previously, along with the response rate and representativeness of the study sample (50% response rate and 49% after data-cleaning) [24]. In brief, a random cluster sampling design at the level of schools, stratified by province and age, was used [24].

Experienced dietitians performed the fieldwork. The school headmasters, teachers and parents were informed about all the study objectives and dietary assessment methods during a school meeting. Oral and written instructions were provided for the recording of foods and drinks consumed by children. Teachers were asked to report what the children consumed at school so that the parents/proxies could include this information in the diaries.

The percentage of underreporters has been described in depth in a previous paper and was shown to be low (<2% when using Goldberg cut-offs adapted for children) [25]. Underreporters were excluded from the study sample for the analyses described in this paper.

The Ethical Committee of the Ghent University Hospital (Belgium) granted ethical approval for the study. All parents of the children participating in the Flanders preschool dietary survey provided their written informed consent.

### 2.2. Dietary Intake Assessment

For the current analyses, only diaries with three completed days were included (*n* = 696; 66% of the collected diaries).

The fiber intake was estimated based on the Belgian NUBEL [26], the Dutch NEVO [27], and the USDA food composition databases [28], which used the enzymatic gravimetric method of the American Association of Analytical Chemists (AOAC) [29,30].

In total, 936 food items and composite dishes were encoded in the original database. All recipes that were described in depth as individual ingredients in the diaries were coded as ingredients. However, in order to classify foods easily into the food groups of the Flemish Food Based Dietary Guidelines (FBDG) [31], eight extra composite dishes had to be disaggregated (nasi goreng, nasi goreng with egg, spaghetti bolognese, chicken ragout, turkey ragout, lasagna, macaroni ham/cheese sauce, and stew). Spaghetti bolognese, for instance, was disaggregated into spaghetti, noodles, minced meat, onions, tomatoes, carrots, and margarine according to the recipe list of the Flemish EPIC-soft version 2004 [32].

After the disaggregating procedures, food items were divided into 57 food groups, based on the classification of the FBDG and the expert opinion of the investigators. It should be noted that, due to lack of information, the complex food mixtures of pizza (consumed by sixty-eight children during the three recorded days) and quiche (consumed by two children) were not disaggregated into their constituent components, but were categorized as a subcategory of the miscellaneous group.

In our study, we defined rest group foods (snacks and desserts) as energy-dense, low-nutritious foods based on the Flemish FBDG, considering their relatively high energy contribution (in Table 2) but low nutrient content.

### 2.3. Socio-Economic Status

Socio-economic status (SES) included family situation (two-parent family, one-parent family or special situation (children living with grandparents or others were considered to be in a special situation)), parental employment (both parents employed, one parent employed or both parents unemployed), and level of parental education (lower secondary education, secondary education or higher education (bachelor, master or above) for both mother and father.

### 2.4. Statistical Analysis

Descriptive statistics of the study population (mean values or frequency distributions and standard deviations (SD)) were calculated by gender-age and gender-SES specific groups. The values of energy and DF intake were corrected for within-person variation by means of the Multiple Source Method (MSM) [33]. The normality of the data and equality of the variances were tested using the Kolmogorov-Smirnov and Levene’s test, respectively. The statistical differences of total energy and (energy-adjusted) DF intake between subgroups were assessed after log-transformation using the Student’s *t*-test. Mean energy-adjusted daily intake from food sources was calculated based on the quartiles of total DF intake. Results were considered statistically significant at an α two-tailed level of 0.05.

The association between DF and SES was investigated by stepwise multiple linear regression analysis, by controlling for potential modifying factors (physical activity level, parental smoking, total energy intake and dietary supplement intake) and confounding factors (gender, age and nationality). Two-way interactions between potential confounding factors and SES were created and examined. In the multiple linear regression analyses, the categories of higher educated mothers, higher educated fathers, unemployed parents and one-parent families, were considered as references. Significance of the associations was evaluated with the *t*-test. Outliers were removed based on residual plots.

Furthermore, to investigate the association between total or major food group-specific DF intakes (bread and cereals, potatoes and grains, vegetables, fruits, and energy-dense, low-nutritious food) and the different independent factors (maternal education level, paternal education level and parental employment), GLM multivariate analyses were carried out with the same references. Other covariates such as potential confounding factors (gender, age and nationality), total energy intake, dietary supplement intake, physical activity, parental smoking, and two-way interactions between SES and confounding factors and between the potential confounding factors, were included in the model.

All statistical analyses were performed using SPSS for Windows version 15.0 (SPSS Inc, Chicago, IL, USA).

## 3. Results

### 3.1. Study Population

A total of 661 out of 1026 children (64%) all with valid information, were included in the analysis (338 boys and 323 girls) (Table 1). Among the 365 excluded children, 330 did not complete 3d EDR days, 51 had a missing value for gender and age, and 4 were missing either gender or age. Out of the 661 children included, 583 children’s height and 609 children’s weight were reported by the parents. As a result, 571 children’s BMI and BMI z-values could be calculated.

The majority of the children (95%) were living with both parents. Approximately half of the parents had a higher education and about 70% of the children’s parents were both employed.

### 3.2. Total Energy and Dietary Fiber Intake

The mean energy intake among preschoolers was 1455 kcal/d (849–2838 kcal/d). Boys had significantly higher energy intakes than girls. The children in the 4–6.5 y group had significantly higher energy intakes than the younger children (*P* ≤ 0.001).

The mean total DF intake of Flemish preschoolers was 13.4 g/d (6.2–21.5 g/d) and the mean energy-adjusted DF intake was 9.3 g/1000 kcal (4.4–17.3 g/1000 kcal) (Table 1). Boys consumed significantly more DF than girls (*P* < 0.001). The elder children consumed more DF than the younger ones (*P* = 0.003). However, energy-adjusted DF intake showed no significant differences between the gender-age groups.

### 3.3. Food Groups Contributing to Dietary Fiber Intake

The most important contributing food groups consisted of bread and cereals (29.5%), particularly bread, rolls, crackers and rice cakes, followed by fruit (17.8%, fresh fruit in particular), potatoes and grains (16.0%, potatoes in particular), energy-dense, low-nutritious foods (12.4%, sweet snacks, french fries and croquettes in particular), and vegetables (11.8%, cooked vegetables in particular) (Table 2).

Additionally, the energy-adjusted daily intakes from bread and cereals, potatoes and grains, vegetables, and fruits increased significantly among the whole population based on the quartiles of total DF intake (*P* < 0.001, *P* = 0.005, *P* < 0.001, and *P* < 0.001, respectively) (Table 3). Energy-adjusted intakes of the rest group, on the other hand, decreased significantly, in boys in particular (*P* < 0.001).

### 3.4. Association between Total and Food-Group Specific Dietary Fiber Intakes and Socio-Economic Status Factor

A significant positive association was observed between children’s total DF intake and one-employed-parent-families (*β* = 0.580, *P* = 0.019), compared to families where both parents were unemployed, whereas a negative association was found with secondary maternal education (*β* = −0.634, *P* = 0.004), as opposed to higher maternal education (Table 4).

GLM multivariate analysis was used to investigate associations between DF intake from main food sources, and SES (Table 5). Compared to children of higher educated mothers, those with a lower secondary maternal education had lower bread and cereal- and rest group, but higher potato and grain-derived fiber intakes (*β* = −8.4, *P =* 0.009, *β* = −4.3, *P =* 0.001, *β* = 8.8, *P <* 0.001, respectively). Conversely, preschoolers of fathers with a secondary education consumed more bread and cereal-, and fruit-derived fibers (*β* = 3.0, *P =* 0.027, *β* = 2.9, *P =* 0.036, respectively) than those with a higher paternal education, whereas children of lower secondary educated fathers had lower potato and grain-derived fiber intakes (*β* = −4.0, *P =* 0.026).

Furthermore, preschoolers’ intake of fiber derived from energy-dense, low-nutritious foods were higher in two-parent families than in one-parent families (*β* = 3.1, *P =* 0.016). Children with one or both parents employed consumed less fibers derived from energy-dense, low-nutritious foods compared to preschoolers of unemployed parents (*β* = −2.8, *P=* 0.010, *β* = −2.6, *P =* 0.012, respectively).

## 4. Discussion

### 4.1. Total and Food Group-Specific Fiber Intake

In this food consumption survey among Belgian preschoolers, the DF intake was on average 13.4 g/d (boys: 13.9 g/d, girls: 12.9 g/d; *P* < 0.001) and the mean energy-adjusted fiber intake 9.3 g/(1000 kcal*d) (boys: 9.2 g/(1000 kcal*d), girls: 9.3 g/(1000 kcal*d); *P* = 0.748). It is noteworthy that a higher energy intake seems to correspond with a higher DF intake in boys, possibly due to a higher overall dietary intake. The mean DF intake among Flemish preschool children did not reach the requirements proposed by the BSHC, especially not for the children aged 4–6.5 y, with 70% of the boys and 81% of the girls not meeting the guidelines.

Compared to the recent small-scale Flemish study of Bosscher *et al.* (2002) (2–3 y old children: 10 g/d based on 7 d-dietary records, *n* = 115), the DF intakes reported in the present study were higher [18]. As limited data is available on Belgian preschoolers’ fiber intake, additional comparisons were made with preschoolers from other countries with comparable age to evaluate our results. The DF intake among Belgian preschoolers were similar to those among European children in general [17], and German (10.3–16.2 g/d) [34] and Italian (11.1–14.6 g/d) [35] children in particular, all assessed by the same dietary assessment method (food diaries). Conversely, the DF intake among Belgian preschoolers were higher than those reported for Spanish (boys: 11.2 g/d, girls: 10.1 g/d) [36] and American children (9.1–13.1 g/d) [37], and lower than those of Swiss children (14.8–16.9 g/d) [38], all based on two 24-h recalls.

Furthermore, this study aimed to identify the most important contributors to total DF intake among preschoolers. However, differences in dietary assessment and, in particular, classification of food items into food groups, often hamper sound comparisons between different study populations. Nevertheless, in general, the main sources of DF were similar for the current study population and children living in Antwerp [18]. However, the latter study reported lower contributions for cereals and pastry (6.6%), fruit (15.1%), and potatoes (14.5%). On the other hand, vegetables (13.9%), soup (8.0%), and sugar and candy products (2.1%) contributed more to the total DF intake than in the more general and representative study population of Flemish preschoolers involved in the present study. Additionally, we found that the group of bread and cereals was the most important contributor of DF, as also observed among American children [39]. Although the contributions of bread and cereals, and vegetables were in line with US reports (29.4% and 11.3%, respectively), potatoes and fruits contributed more to the DF intake of Belgian preschoolers than of American children (11.2% and 13.1%, respectively) [39]. In comparison to Spanish children [40], the contributions of bread and cereals, potatoes, and vegetables were lower in Spanish children (11.2%, 4.3%, and 7.9%, respectively) than in ours, while those of fruit and legumes were much higher in Spanish children (25.6% and 20.1%, respectively) than in Belgian children. Finally, the average DF intakes from cereals, fruit, and vegetables were substantially lower in Belgian than German children (4.4–8.0 g/d, 2.8–3.3 g/d, and 2.4–3.0 g/d, respectively) [34].

When looking at the food groups that are being under-consumed according to the FBDG [41] and, taking into account the contributions of these foods to the total fiber intake in these preschoolers, it can be concluded that higher intakes of whole-wheat bread, fruit, and vegetables, could significantly increase the fiber intake and should, therefore, be promoted among preschoolers.

### 4.2. Associations of Fiber Intake with Socio-Economic Status

To the best of our knowledge, there is no data available on possible associations between DF intake and SES factors among Belgian children. Our results indicate that children of secondary educated mothers have lower DF intake than those of higher educated mothers, whereas children with one parent being employed consumed more DF compared to those with unemployed parents. Similarly, maternal and paternal level of education were related to the food group-specific fiber intake of their children, with lower bread and cereal-, higher potato and grain-, and lower energy-dense, low-nutritious foods-derived fiber intake reported for children of lower secondary educated mothers compared to those of higher educated mothers. On the other hand, higher bread and cereal-, and fruit-derived fiber intake was observed with paternal secondary education as opposed to higher education. Additionally, children with employed parents had higher total DF intakes, but consumed less DF from energy-dense, low-nutritious food than preschoolers with both parents unemployed. In two-parent families, children had higher intake of energy-dense, low-nutritious food-derived fibers than in one-parent families.

Perry *et al.* (1988) suggested that parental involvement plays a critical role in promoting children’s health behavior and dietary habits at an early age [42]. Parental involvement might result in consumption of fiber from high-nutritious foods (vegetables and fruit). In the present study, DF intake, more from high-nutritious foods (vegetables and fruit) and less from energy-dense, low-nutritious foods, were reported for preschoolers of higher educated mothers. Also, evidence showed that children in low SES families were found to have higher total energy, cholesterol, and fat intake and lower vegetable and fruit intake [43–47]. Moreover, children of unemployed parents or lesser income families consumed unhealthier DF, derived from energy-dense, low nutritious foods. The cost of healthy food, reduced food choices, and lack of education in low SES families might lead to lower vegetable- and fruit-derived fiber intake and, consequently, a higher prevalence of children at risk to become overweight or obese, and to develop chronic diseases [20,23,48]. Children with both employed parents, however, had less DF intake than those with one-employed parent in our study, which might be influenced by parents having less free time.

We observed that dietary sources from vegetables and fruit contributing to DF intake in our study were much less compared to other food sources based on the quartiles of total fiber intake. Although vegetables and fruit were ranked second and fifth in DF contribution, children had extremely lower DF intake from raw vegetables (1.8%) compared to cooked ones (10.0%). In addition, in our findings, children of higher educated mothers and secondary educated fathers and those with one or both parents being employed had more DF intake from vegetables and fruit, which indicates that lower secondary educated and unemployed families need to be targeted during health promotion campaigns. Our results also suggest that the level of maternal education is more indicative for dietary habits of their preschool aged children than the level of paternal education.

### 4.3. Strengths and Limitations

The present study was the first food consumption survey among preschoolers comparing associations between total and food group-specific DF intakes and SES in Belgium while covering the whole Flemish region. Therefore, the results of this large cross-sectional study represent the Flemish preschool children’s dietary habits with a good representation compared to the more local and small-scale surveys that were executed before.

Like all studies, some limitations should be taken into consideration. First, this study suffered from some selection bias, with the lower SES group being underrepresented [24], which might have influenced the true DF intake and the linear associations.

Furthermore some limitations regarding the dietary assessment method are noteworthy. No dietary assessment method is perfect and every method is prone to some degree of misreporting. The method of 3d EDR reflects the individual children’s short-term rather than usual intake. However, we corrected for within-person variability by using the MSM method to obtain a more precise individual usual daily DF intake. The percentage of under-reporters, excluded in this study, in the final sample for analysis was very low (2%). In addition, a relative validation study was conducted in which the results derived from a food frequency questionnaire were compared with those derived from our 3d EDR for calcium intake, food intake and for a diet quality index [41,49,50].

Moreover, the decisions regarding the food grouping were based on the Flemish FBDG and on the judgment of the investigators, which might have implications for the findings. The food composition of fortified foods, highly consumed by Flemish preschoolers, was rather hard to define and, in some cases, information from the industry or from packing materials had to be used. Furthermore, the definition of DF in Belgium is considered as carbohydrates with three to ten monomeric units, which might result in differences with international recommendations [51]. Also, no real information is available on the low molecular weight DF fraction and limitations of AOAC methods used [52,53]. Our dataset was not adjusted for possible alterations in fiber content or quality due to food processing, which may have attenuated the accuracy of our total DF estimates.

## 5. Conclusions

Our results showed that the mean total DF intake among preschoolers is below the guidelines of the BSHC, especially for children aged 4–6.5 y. Girls ingested significantly less fibers than boys. The most important contributor to the total DF intake was the group of bread and cereals, followed by fruit, potatoes, energy-dense, low nutritious foods, and vegetables. Maternal education level and parental employment were significantly associated with DF intake. Overall, DF intakes from high-nutritious foods (vegetables and fruit) were higher in preschoolers of higher educated mothers and those with one or both parents employed. These findings suggest that dietary fiber should be promoted in general and low SES families should be addressed in particular.

## Figures and Tables

**Table 1.** Anthropometric characteristics and socio-economic status (*n* and %), and energy and (energy-adjusted) dietary fiber intakes ^μ^ reported for Flemish preschool children.

**Characteristic**	**Total**	**Boys**	**Girls**
**Age (*n* = 661)**	*n*(%)		
2.5–4 y	197 (29.8)	102 (30.2)	95 (29.4)
4–6.5 y	464 (70.2)	236 (69.8)	228 (70.6)
**Socio-economic status**			
**Family situation (*n*= 659)**			
Two-parent family	632 (95.9)	323 (96.1)	309 (95.7)
One-parent family	23 (3.5)	11 (3.3)	12 (3.7)
Special situation	4 (0.6)	2 (0.6)	2 (0.6)
**Maternal education (*n*= 655)**			
Lower secondary	26 (4.0)	9 (2.7)	17 (5.3)
Secondary	250 (38.2)	127 (38.1)	123 (38.2)
Higher education	379 (57.9)	197 (59.2)	182 (56.5)
**Paternal education (*n*= 637)**			
Lower secondary	49 (7.7)	25 (7.7)	24 (7.7)
Secondary	279 (43.8)	146 (45.1)	133 (42.5)
Higher education	309 (48.5)	153 (47.2)	156 (49.8)
**Parental employment (*n*= 634)**			
Both parents employed	439 (69.2)	214 (66.0)	225 (72.6)
One parent employed	157 (24.8)	88 (27.2)	69 (22.3)
Unemployed parents	38 (6.0)	22 (6.8)	16 (5.2)
	Mean intake ± SD		
	Total energy intake (kcal/d)		
2.5–4 y *	1408.4 ± 260.4	1441.7 ± 253.0	1372.7 ± 264.9
4–6.5 y **	1474.4 ± 240.0 ^a^	1526.3 ± 233.7 ^b^	1420.8 ± 235.1
	Energy-adjusted fiber intake (g/(1000 kcal * d))		
2.5–4 y	9.2 ± 1.8	9.4 ± 1.8	9.0 ± 1.9
4–6.5 y	9.3 ± 1.9	9.3 ± 1.9	9.3 ± 1.8
	Total fiber intake (g/d)		
2.5–4 y *	12.9 ± 3.0	13.4 ± 3.0	12.2 ± 2.9
4–6.5 y *	13.7 ± 3.2 ^b^	14.1 ± 3.3	13.2 ± 3.2 ^b^

SD: standard deviation.

μ Mean daily dietary fiber intake was calculated and adjusted for within-person variation using Multiple Source Method (MSM).

* Mean value was significantly different between boys and girls, Student *t*-test after log-transformation, *P* < 0.05.

** Mean value was significantly different between boys and girls, Student *t*-test after log-transformation, *P* ≤ 0.001.

a Mean value was significantly different from 2.5–4 y old, Student *t*-test after log-transformation, *P* ≤ 0.001.

b Mean value was significantly different from 2.5–4 y old, Student *t*-test after log-transformation, *P* < 0.05.

**Table 2.** Mean and median daily intakes of food groups ^μ^ and their contributions to total energy and fiber intakes (*n* = 661).

**Food Group**	**Food Intake (g/d)**			**Energy**		**Fiber**	
	**Mean**	**Median**	***SD***	**%**	**Order**	**%**	**Order**
**Beverages (including juices, excluding the rest group)**	486.2			5.2		4.8	
Water	224.2	150.0	(226.4)	0.0		0.0	
Light beverages	23.1	0.0	(90.1)	0.0		0.0	
Tea and coffee without sugar	8.2	0.0	(43.5)	0.0		0.1	
Fruit juice	172.8	150.0	(209.3)	4.5	6	2.9	8
Vegetable juice	0.2	0.0	(6.0)	0.0		0.0	
Soup, bouillon	57.7	0.0	(101.7)	0.6		1.8	
**Bread and cereals**	86.7			16.4		29.5	
Bread, rolls, crackers, rice cakes	70.3	62.5	(46.8)	12.4	1	25.3	1
Sugared bread	7.5	0.0	(22.5)	1.7		2.2	9
Breakfast cereals (ready-to-eat, hot)	8.9	0.0	(20.0)	2.3		2.0	
**Potatoes and grains**	86.7			5.4		16.0	
Pasta, noodles	15.4	0.0	(41.0)	1.1		1.0	
Rice	6.3	0.0	(25.5)	0.6		0.9	
Potatoes	65.0	50.0	(69.3)	3.7	7	14.1	3
**Vegetables**	66.5			1.1		11.8	
Cooked vegetables	53.7	40.0	(60.1)	1.0		10.0	4
Raw vegetables	12.8	0.0	(38.3)	0.1		1.8	
**Fruits (sweetened and unsweetened)**	109.9			4.4		17.8	
Fresh fruit	94.0	68.8	(102.7)	3.6	8	15.5	2
Canned fruit	15.4	0.0	(45.4)	0.7		2.1	10
Dried fruit	0.4	0.0	(3.7)	0.1		0.2	
Olives	0.1	0.0	(1.5)	0.0		0.0	
**Milk, milk products, and calcium enriched soy drinks**	439.9			19.9		6.0	
Milk (including goat’s milk)	179.0	125.0	(218.5)	6.2	4	0.0	
Flavoured milk drinks (e.g., Fristi, chocolate milk…)	188.3	145.0	(226.8)	8.9	3	4.5	6
Yoghurt	4.5	0.0	(25.3)	0.2		0.0	
Sugared or aromatised yoghurt	14.2	0.0	(46.9)	0.9		0.2	
Soy drinks	15.7	0.0	(82.5)	0.6		1.0	
Milk desserts	19.9	0.0	(56.2)	1.7		0.2	
Soy-based desserts	2.3	0.0	(19.1)	0.1		0.1	
Fermented milk or soy drinks (e.g., actimel, yakult…)	0.7	0.0	(7.4)	0.0		0.0	
Fresh cheese	15.3	0.0	(43.3)	1.4		0.0	
**Cheese**	14.5			3.5		0.0	
Hard cheese (no cream cheese)	11.8	0.0	(22.6)	3.0		0.0	
Cheese spread	2.7	0.0	(8.8)	0.5		0.0	
**Fat, oil, cream cheese, sour cream**	8.6			3.3		0.0	
Butter, margarine	8.3	6.0	(9.5)	3.1		0.0	
Oil	0.3	0.0	(1.4)	0.2		0.0	
Frying oil	0.0	0.0	(0.6)	0.0		0.0	
**Meat, poultry, fish, eggs, vegetarian products**	90.3			13.5		1.3	
Meat, game, meat products	37.2	20.0	(46.1)	6.0	5	0.1	
Chicken, turkey	15.9	0.0	(34.7)	1.9		0.0	
Fish, shellfish	8.5	0.0	(28.7)	0.9		0.1	
Cold cuts from meat products	20.7	6.8	(30.2)	3.5	9	0.0	
Cold cuts from fish products	0.9	0.0	(6.8)	0.2		0.0	
Eggs^†^	5.1	0.0	(18.2)	0.7		0.0	
Vegetarian products (e.g., tofu, tempé…)	1.7	0.0	(11.6)	0.2		0.9	
Nuts and seeds	0.3	0.0	(3.4)	0.1		0.2	
**Rest group (snacks and desserts)^a^**	201.8			26.8		12.4	
Brioches	3.5	0.0	(17.0)	0.8		0.6	
Sweet snacks	43.6	32.0	(43.5)	11.9	2	5.2	5
Salty snacks	2.1	0.0	(9.8)	0.8		0.9	
Tea and coffee with sugar	3.2	0.0	(26.6)	0.0		0.0	
Soft drinks	97.7	0.0	(169.4)	2.7		0.0	
Salty sauces	12.5	0.0	(24.9)	1.6		0.6	
Cream	0.3	0.0	(2.6)	0.1		0.0	
Sweet sauces	0.1	0.0	(2.5)	0.0		0.0	
Chocolate	3.1	0.0	(9.5)	1.1		0.2	
Chocolate spread	9.4	0.0	(13.9)	3.5	10	1.1	
Other sweet spread (e.g., jam, honey…)	5.3	0.0	(11.6)	1.0		0.3	
Sugar	0.1	0.0	(0.9)	0.0		0.0	
Fried snacks	0.1	0.0	(2.6)	0.0		0.0	
French fries, croquettes	14.6	0.0	(37.7)	2.6		3.5	7
Sweet desserts (e.g., ice cream, tiramisu…)	6.2	0.0	(23.2)	0.8		0.1	
**Miscellaneous**	4.2			0.5		0.3	
Pizza and quiches	2.2	0.0	(17.8)	0.3		0.2	
Other miscellaneous ^‡^	2.0	0.0	(21.3)	0.2		0.1	

μ These mean food group intakes are rough estimates calculated from the raw data on which these nutrient contributions are based, without adjustment for within-person variation. The high number of non-consumers in some food groups hindered the adjustment for within-person variation.

† Includes only eggs reported separately and eggs included in disaggregated food mixtures.

‡ Includes foods or components with negligible contributions to the total nutrient intakes that could not be categorized in the above food groups (e.g., herbs and spices, monosodium glutamate, starch, plain gelatin, artificial sweeteners, pectin, cocoa powder…).

a Rest group (snacks and desserts) was defined as energy-dense, low-nutritious foods.

**Table 3.** Mean energy-adjusted daily intakes (g/d) from the main food groups contributing to dietary fiber intake for the children assigned to the different total dietary fiber intake quartiles (*n* = 661).

	**Fiber Intake Quartiles**												***P***^†^		
	**Total**				**Boys**				**Girls**						

**Food Groups**^μ^	***Q*1**	***Q*2**	***Q*3**	***Q*4**	***Q*1**	***Q*2**	***Q*3**	***Q*4**	***Q*1**	***Q*2**	***Q*3**	***Q*4**	**Total**	**Boys**	**Girls**
Bread and cereals	51.6	57.5	62.6	66.7	53.0	57.6	61.9	68.0	50.6	57.4	63.7	64.7	<0.001	<0.001	<0.001
Potatoes and grains	53.5	59.1	63.3	64.1	59.1	59.9	63.4	57.3	49.2	58.5	63.3	74.8	0.005	0.725	<0.001
Vegetables	33.1	42.3	47.0	60.1	33.2	42.5	46.1	56.4	33.0	42.1	48.2	65.9	<0.001	<0.001	<0.001
Fruits	41.2	63.3	83.2	110.4	33.5	54.0	80.7	106.6	47.3	70.4	86.4	116.3	<0.001	<0.001	<0.001
Rest group^‡^	123.3	112.5	93.5	82.4	143.2	140.7	89.7	87.8	107.9	90.8	98.3	73.9	<0.001	<0.001	0.178

μ Quartiles based on total fiber intake among Flemish children.

† Statistical analysis was tested by ANOVA (bread and cereals, potatoes and grains) and Kruskal-Wallis (vegetables, fruits and Rest group) test.

‡ Rest group (snacks and desserts) was defined as energy-dense, low-nutritious foods.

**Table 4.** Stepwise multiple linear regression analysis of the potential association between total dietary fiber intake and socio-economic status among Flemish preschoolers (*n* = 661).

	*β*	***SE***	**95%*CI***	***P***

**Total fiber intake**^†^^‡^				
Secondary maternal education ^μ^	−0.634	0.219	−1.1, −0.203	0.004
One employed parent ^μ^	0.580	0.247	0.095, 1.1	0.019
Age * two-parent family ^μ^	0.212	0.097	0.021, 0.403	0.030

SE: standard error of *β* coefficient; *CI*: confidence interval.

μ Higher maternal education, and two-unemployed–parent-families and one-parent families were used as reference categories.

† Adjusted for total energy intake, age, gender, nationality, and children’s level of physical activity, parental lifestyle and interactions.

‡ Non-significant variables with standardized coefficients *β*.

Higher maternal education: *β* = 0.125, *P* = 0.148; Secondary paternal education: *β* = −0.037, *P* = 0.297; Higher paternal education: *β* = 0.038, *P* = 0.303; Both employed parent: *β* = −0.040, *P* = 0.547; Two-parent family: *β* = 0.010, *P* = 0.797.

**Table 5.** GLM multivariate analysis of the potential association between food group-specific fiber intake and socio-economic status of Flemish preschoolers (*n* = 661).

	**Bread and Cereals**			**Potatoes and Grains**			**Vegetables**			**Fruits**			**Rest Group**†		
**Independent variables**^μ^	*β***(*SE*)**	**95%*CI***	***P***	*β***(*SE*)**	**95%*CI***	***P***	*β***(*SE*)**	**95%*CI***	***P***	*β***(*SE*)**	**95%*CI***	***P***	*β***(*SE*)**	**95%*CI***	***P***
**Maternal education**															
Lower secondary	−8.4 (3.2)	−14.7, −2.1	0.009	8.8 (2.2)	4.4, 13.1	<0.001	2.5 (2.2)	−1.9, 6.8	0.262	−1.7 (3.3)	−8.2, 4.8	0.613	−4.3 (1.3)	−7.0, −1.7	0.001
Secondary	−2.2 (1.4)	−4.9, −0.42	0.098	0.16 (0.94)	−2.0, 1.7	0.862	−0.016 (0.94)	−1.9, 1.8	0.974	−1.9 (1.4)	−4.6, 0.88	0.183	0.082 (0.57)	−1.0, 1.2	0.885
**Paternal education**															
Lower secondary	3.9 (2.6)	−1.2, 8.9	0.131	−4.0 (1.8)	−7.5, −0.48	0.026	−0.41 (1.8)	−3.9, 3.1	0.819	−1.2 (2.7)	−6.5,4.0	0.640	1.5 (1.1)	−0.61, 3.7	0.160
Secondary	3.0 (1.3)	0.33, 5.6	0.027	−1.0 (0.94)	−2.8, 0.82	0.862	0.39 (0.93)	−1.4, 2.2	0.676	2.9 (1.4)	0.20, 5.7	0.036	0.73 (0.57)	−0.39, 1.8	0.201
**Family situation**															
Two-parent	2.3 (3.0)	−3.7, 8.2	0.439	1.9 (2.1)	−2.2, 5.9	0.372	1.8 (2.1)	−2.3, 5.9	0.392	2.3 (3.1)	−3.8, 8.4	0.464	3.1 (1.3)	0.58, 5.5	0.016
**Parental employment**															
Both employed	−1.4 (2.4)	−6.1, 3.4	0.577	−0.89 (1.7)	−4.2, 2.4	0.600	1.4 (1.7)	−2.0, 4.7	0.424	−2.9 (2.5)	−7.8, 2.1	0.254	−2.6 (1.0)	−4.6,−0.56	0.012
One employed	−1.2 (2.6)	−6.3, 3.9	0.653	−0.061 (1.8)	−3.6, 3.5	0.973	2.0 (1.8)	−1.6, 5.5	0.272	−2.3 (2.7)	−7.5, 3.0	0.399	−2.8 (1.1)	−5.0, −0.67	0.010

SE: standard error of *β* coefficient; *CI*: confidence interval.

μ Unemployed parents, higher educated parents and one-parent family were as reference.

† Rest group (snacks and desserts) was defined as energy-dense, low-nutritious foods.

## References

[1] Chandalia M (2000). Beneficial effects of high dietary fiber intake in patients with type 2 diabetes mellitus. N. Engl. J. Med.

[2] Nakaji S (2002). Trends in dietary fiber intake in Japan over the last century. Eur. J. Nutr.

[3] WHO (World Health Organization) (2003). Diet, nutrition, and the prevention of chronic diseases. WHO Tech. Rep. Ser..

[4] Cummings JH, Mann JI, Nishida C, Vorster HH (2009). Dietary fibre: an agreed definition. Lancet.

[5] Affenito SG, Thompson DR, Barton BA, Franko DL, Daniels SR, Obarzanek E, Schreiber GB, Striegel-Moore RH (2005). Breakfast consumption by African-American and white adolescent girls correlates positively with calcium and fiber intake and negatively with body mass index. J. Am. Diet. Assoc.

[6] Butt MS, Shahzadi N, Sharif MK, Nasir M (2007). Guar gum: A miracle therapy for hypercholesterolemia, hyperglycemia and obesity. Crit. Rev. Food Sci. Nutr.

[7] Davis JN, Alexander KE, Ventura EE, Toledo-Corral CM, Goran MI (2009). Inverse relation between dietary fiber intake and visceral adiposity in overweight Latino youth. Am. J. Clin. Nutr.

[8] Du H, van der AD, Boshuizen HC, Forouhi NG, Wareham NJ, Halkjaer J, Tjonneland A, Overvad K, Jakobsen MU, Boeing H (2010). Dietary fiber and subsequent changes in body weight and waist circumference in European men and women. Am. J. Clin. Nutr.

[9] He J, Klag MJ, Whelton PK, Mo JP, Chen JY, Qian MC, Mo PS, He GQ (1995). Oats and buckwheat intakes and cardiovascular disease risk factors in an ethnic minority of China. Am. J. Clin. Nutr.

[10] Federal Public Service for Public Health and Food-Chain Safety and Environment (2009). Dietary Recommendations for Belgium.

[11] WHO Population nutrient intake goals for preventing diet-related chronic diseases. http://www.who.int/dietphysicalactivity/-publications/trs916/en/gsfao_overall.pdf.

[12] US Department of Agriculture, National Agricultural Library and National Academy of Sciences, Institute of Medicine & Food and Nutrition Board (2005). Dietary Reference Intakes for Energy, Carbohydrate, Fiber, Fat, Fatty Acids, Cholesterol, Protein, and Amino Acids (Macronutrients).

[13] British Nutrition Foundation Dietary fibre. http://www.nutrition.org.uk/-nutritionscience/nutrients/dietary-fibre?start=4.

[14] Francis CC, Bope AA, MaWhinney S, Czajka-Narins D, Alford BB (1999). Body composition, dietary intake, and energy expenditure in nonobese, prepubertal children of obese and nonobese biological mothers. J. Am. Diet. Assoc.

[15] Ruottinen S, Lagstrom HK, Niinikoski H, Ronnemaa T, Saarinen M, Pahkala KA, Hakanen M, Viikari JS, Simell O (2010). Dietary fiber does not displace energy but is associated with decreased serum cholesterol concentrations in healthy children. Am. J. Clin. Nutr.

[16] Williams CL, Bollella M (1995). Is a high-fiber diet safe for children?. Pediatrics.

[17] Lambert J, Agostoni C, Elmadfa I, Hulshof K, Krause E, Livingstone B, Socha P, Pannemans D, Samartin S (2004). Dietary intake and nutritional status of children and adolescents in Europe. Br. J. Nutr.

[18] Bosscher D, van Caillie-Bertrand M, Deelstra H (2002). Daily dietary fibre intake of children, 2 to 3 years of age, living in Antwerp, Belgium. Nutr. Res.

[19] Albertson AM, Affenito SG, Bauserman R, Holschuh NM, Eldridge AL, Barton BA (2009). The relationship of ready-to-eat cereal consumption to nutrient intake, blood lipids, and body mass index of children as they age through adolescence. J. Am. Diet. Assoc.

[20] Hulshof KF, Brussaard JH, Kruizinga AG, Telman J, Lowik MR (2003). Socio-economic status, dietary intake and 10 y trends: the Dutch National Food Consumption Survey. Eur. J. Clin. Nutr.

[21] Johnson L, Mander AP, Jones LR, Emmett PM, Jebb SA (2008). Energy-dense, low-fiber, high-fat dietary pattern is associated with increased fatness in childhood. Am. J. Clin. Nutr.

[22] Langlois K, Garriguet D, Findlay L (2009). Diet composition and obesity among Canadian adults. Health Rep.

[23] Wilson TA, Adolph AL, Butte NF (2009). Nutrient adequacy and diet quality in non-overweight and overweight Hispanic children of low socioeconomic status: the Viva la Familia Study. J. Am. Diet. Assoc.

[24] Huybrechts I, Matthys C, Pynaert I, De Mayer M, Bellemans M, De Geeter H, De Henauw S (2008). Flanders preschool dietary survey: Rationale, aims, design, methodology and population characteristics. Arch. Public Health.

[25] Huybrechts I, De Henauw S (2007). Energy and nutrient intakes by pre-school children in Flanders-Belgium. Br. J. Nutr.

[26] NUBEL (2004). Belgium Food Composition Table.

[27] NEVO (2003). Dutch Food Composition Table 2001.

[28] US Department of Agriculture (USDA), US Department of Health and Human Services (2005). Dietary Guidelines for Americans.

[29] Butler R, Patel J (2010). A direction in dietary fibre analysis. Nutr. Food Sci.

[30] McCleary BV, DeVries JW, Rader JI, Cohen G, Prosky L, Mugford DC, Champ M, Okuma K (2010). Determination of total dietary fiber (CODEX definition) by enzymatic-gravimetric method and liquid chromatography: Collaborative study. J. AOAC Int.

[31] VIG (2004). De voedingsdriehoek: een Praktische Voedingsgids.

[32] De Vriese S, Huybrechts I, Moreau M, van Oyen H (2006). Enquête de Consommation Alimentaire Belge 1–2004: Rapport.

[33] Department of Epidemiology of the German Institute of Human Nutrition Potsdam-Rehbrücke Multiple Source Method (MSM). https://nugo.dife.de/msm/.

[34] Buyken AE, Cheng G, Gunther AL, Liese AD, Remer T, Karaolis-Danckert N (2008). Relation of dietary glycemic index, glycemic load, added sugar intake, or fiber intake to the development of body composition between ages 2 and 7 y. Am. J. Clin. Nutr.

[35] Grammatikopoulou MG, Daskalou E, Hatzopoulou M, Sourtzinou L, Tsigga M (2009). Comparing diet composition and growth of children living in two limitary Greek islands (Samos and Corfu). Public Health Nutr.

[36] Serra-Majem L, Ribas-Barba L, Perez-Rodrigo C, Bartrina JA (2006). Nutrient adequacy in Spanish children and adolescents. Br. J. Nutr.

[37] Alaimo K, McDowell MA, Briefel RR, Bischof AM, Caughman CR, Loria CM, Johnson CL, Alaimo K, McDowell MA, Briefel RR (1994). Dietary intake of vitamins, minerals, and fiber of persons ages 2 months and over in the United States: Third National Health and Nutrition Examination Survey, Phase 1, 1988–91Third National Health and Nutrition Examination Survey, Phase 1, 1988–91. Adv. Data.

[38] Aeberli I, Kaspar M, Zimmermann MB (2007). Dietary intake and physical activity of normal weight and overweight 6 to 14 year old Swiss children. Swiss Med. Wkly.

[39] Saldanha LG (1995). Fiber in the diet of US children: results of national surveys. Pediatrics.

[40] Royo-Bordonada MA, Gorgojo L, de Oya M, Garces C, Rodriguez-Artalejo F, Rubio R, del Barrio JL, Martin-Moreno JM (2003). Food sources of nutrients in the diet of Spanish children: the Four Provinces Study. Br. J. Nutr.

[41] Huybrechts I, Matthys C, Vereecken C, Maes L, Temme EH, van Oyen H, De Backer G, de Henauw S (2008). Food intakes by preschool children in Flanders compared with dietary guidelines. Int. J. Environ. Res. Public Health.

[42] Perry CL, Luepker RV, Murray DM, Kurth C, Mullis R, Crockett S, Jacobs DR (1988). Parent involvement with children's health promotion: the Minnesota Home Team. Am. J. Public Health.

[43] Casey PH, Szeto K, Lensing S, Bogle M, Weber J (2001). Children in food-insufficient, low-income families: prevalence, health, and nutrition status. Arch. Pediatr. Adolesc. Med.

[44] Drewnowski A, Specter SE (2004). Poverty and obesity: the role of energy density and energy costs. Am. J. Clin. Nutr.

[45] Drewnowski A, Darmon N (2005). Food choices and diet costs: an economic analysis. J. Nutr.

[46] Knol LL, Haughton B, Fitzhugh EC (2005). Dietary patterns of young, low-income US children. J. Am. Diet. Assoc.

[47] Langevin DD, Kwiatkowski C, McKay MG, Maillet JO, Touger-Decker R, Smith JK, Perlman A (2007). Evaluation of diet quality and weight status of children from a low socioeconomic urban environment supports “at risk” classification. J. Am. Diet. Assoc.

[48] Doyle W, Jenkins S, Crawford MA, Puvandendran K (1994). Nutritional status of schoolchildren in an inner city area. Arch. Dis. Child.

[49] Huybrechts I, De Bacquer D, Matthys C, De Backer G, De Henauw S (2006). Validity and reproducibility of a semi-quantitative food-frequency questionnaire for estimating calcium intake in Belgian preschool children. Br. J. Nutr.

[50] Huybrechts I, Vereecken C, de Bacquer D, Vandevijvere S, Van Oyen H, Maes L, Vanhauwaert E, Temme L, de Backer G, De Henauw S (2010). Reproducibility and validity of a diet quality index for children assessed using a FFQ. Br. J. Nutr.

[51] Howlett JF, Betteridge VA, Champ M, Craig SA, Meheust A, Jones JM (2010). The definition of dietary fiber-discussions at the Ninth Vahouny Fiber Symposium: building scientific agreement. Food Nutr Res.

[52] Cowin I, Emmett P (1999). The effect of missing data in the supplements to McCance and Widdowson’s food tables on calculated nutrient intakes. Eur. J. Clin. Nutr.

[53] Post BE, Marshak MR, DeVries JW (2010). Simultaneous ion removal and quantitation of low-molecular-weight dietary fiber from high-molecular-weight dietary fiber filtrates using liquid chromatography. J. AOAC Int.

